# Integration of ternary I-III-VI quantum dots in light-emitting diodes

**DOI:** 10.3389/fchem.2023.1106778

**Published:** 2023-03-23

**Authors:** Nery Islas-Rodriguez, Raybel Muñoz, Jose A. Rodriguez, Rosa A. Vazquez-Garcia, Martin Reyes

**Affiliations:** ^1^ Universidad Autonoma del Estado de Hidalgo (UAEH). Area Academica de Ciencias de La Tierra y Materiales, Hgo, Mexico; ^2^ Universidad Autonoma del Estado de Hidalgo (UAEH). Area Academica de Quimica, Hidalgo, Mineral de la Reforma, Mexico

**Keywords:** Ternary I-III-VI quantum dots, synthesis methods, physicochemical properties of TQDs, light-emitting diodes, white light-emitting diodes

## Abstract

Ternary I-III-VI quantum dots (TQDs) are semiconductor nanomaterials that have been gradually incorporated in the fabrication of light-emitting diodes (LEDs) over the last 10 years due to their physicochemical and photoluminescence properties, such as adequate quantum yield values, tunable wavelength emission, and easy synthesis strategies, but mainly because of their low toxicity that allows them to be excellent candidates to compete with conventional Cd-Pb-based QDs. This review addresses the different strategies to obtain TQDs and how synthesis conditions influence their physicochemical properties, followed by the LEDs parameters achieved using TQDs. The second part of the review summarizes how TQDs are integrated into LEDs and white light-emitting diodes (WLEDs). Furthermore, an insight into the state-of-the-art LEDs development using TQDs, including its advantages and disadvantages and the challenges to overcome, is presented at the end of the review.

## Introduction

For several years, lighting has constituted 19% of global electrical energy consumption; consequently, the use of light-emitting diodes (LEDs) is considered useful for energy savings as a result of properties such as their long lifespan and high luminous efficiency (Chen T. et al., 2019). Therefore, they have been progressively replacing traditional lighting and are now well established in the luminosity market (Taki and Strassburg, 2020). General applications of LEDs involve white light-emitting diodes (WLEDs), indicator lights for electronic circuits (Bui and Hauser, 2015), and fluorescence sensing, among others (Mukunda et al., 2022).

On the other hand, the existence of colloidal nanocrystals of semiconductor materials known as quantum dots (QDs) has facilitated their integration into LEDs fabrication due to their notable photoluminescence quantum yield, photochemical stability, wide absorption, and narrow emission spectra (Liu and Su, 2014; Cao and An, 2015; Zhang et al., 2015; Su et al., 2020; Muñoz et al., 2021; Chen et al., 2022).

The contribution of QDs based on Cd-Pb for the preparation of high-performance LEDs has been remarkable. However, the inherent toxicity of these elements causes negative effects in human health and the environment, restricting their commercialization (Tsolekile et al., 2017; Li et al., 2018; Kim et al., 2020). As a potential alternative, the use of ternary I-III-VI quantum dots (TQDs) has gained growing interest because of their lower toxicity, easy processing, lower cost, and tunable photoluminescence emission. In addition, compared to their binary analogues, TQDs have a higher degree of compositional flexibility and size control. For example, CuInS_2_, CuGaS_2_, and AgInS_2_ nanomaterials have been recently applied to the construction of LEDs (Mei et al., 2018; Wang et al., 2019; Su et al., 2020; Oluwafemi et al., 2021; May et al., 2022; Wei et al., 2022). This review describes the synthesis, physicochemical properties, and application of TQDs for LEDs production and includes a perspective of LEDs development using TQDs.

## Methods for the synthesis of TQDs

The methods for TQDs synthesis can be classified according to the solvent used in the reaction. In this sense, several organic- and aqueous-based strategies have been developed to obtain TQDs with specific physicochemical properties depending on their final application.

TQDs synthesis requires at least three main components: the ion precursors that form the nanostructure, the stabilizing agent, and the solvent. In TQDs synthesis, two cations (one from group I such as Cu^+^, Ag^+^, or Au^+^ and one from group III such as In^3+^, Ga^3+^, or Al^3+^) are needed, together with one anion from group VI (S^2-^ or Se^2-^) (Tsolekile et al., 2017). The nature of the ion precursors depends on the solvent used, and the ions source can be organic (acetates or diethyldithiocarbamate) or inorganic (nitrates or chlorides) salts (Huang et al., 2020; Su et al., 2020; Ming et al., 2021). The second component is the stabilizing agent, also called the ligand, which is an organic molecule that allows to obtain TQDs dispersed in the solvent by bonding to the cations on the TQDs surface and establishing an equilibrium between the chain and the solvent. Organic synthesis of TQDs employs surfactants as ligands with one polar head and one or several hydrophobic carbon chains. The main ligands used in TQDs synthesis are 1-dodecanthiol, trioctylphosphine oxide, oleylamine, and octadecylamine for organic synthesis and thiol-containing ligands (such as l-cysteine or 3-mercaptopropionic acid) for aqueous synthesis (Hu et al., 2018; Jain et al., 2020).

In addition to TQDs, other structures have been developed to enhance the physicochemical properties of the nanoparticle, namely, core/shell and core/shell/shell TQDs. Shell structures are commonly composed of ZnS or ZnSe deposited over the core structure, and a third cation (Zn^2+^) and an excess of S^2-^ or Se^2-^ are used in the synthesis to obtain the shell structure (Tsolekile et al., 2020). To ensure the synthesis of a core/shell structure, the precursor of the Zn^2+^ ion is added after complete formation of the core to avoid the synthesis of quaternary compounds. As in core obtention, Zn^2+^ cation precursors can be inorganic coordination precursors in organic solvents for organic synthesis or a salt (nitrate or chloride) for aqueous synthesis (Miropoltsev et al., 2020).

As previously mentioned, the composition and synthesis conditions of TQDs will depend on their final application, and for LEDs fabrication, the most common compounds are AgInS_2_ and CuInS_2_. In this review, the different strategies for the synthesis of AgInS_2_, CuInS_2_, and other TQDs are presented and discussed. The classification of the synthesis strategies is based on the solvent nature (organic or aqueous).

### Organic synthesis

Organic synthesis allows to obtain high-quality TQDs. The processes generally involve high temperatures (≥100°C), the use of inorganic coordination precursors (acetates and oleates), and hydrophobic ligands dissolved in organic solvents. Furthermore, inert atmospheres are often required due to the susceptibility of reagents to air oxygen (May et al., 2022). The main approaches include solvothermal, hot injection, heating-up, and thermal decomposition methods.

### Solvothermal method

The solvothermal method refers to a synthetic strategy where the temperature of the reaction takes place above the boiling point of the solvent by increasing pressure in the reaction system. This method has some advantages when it is used for TQDs synthesis. By using an autoclave, the heat convection is more homogeneous, leading to a narrow size distribution of TQDs (Kashyap et al., 2021). Furthermore, a higher pressure promotes the crystallization process, which, combined with the relatively mild reaction conditions, allows to control the morphological parameters (shape and size) and avoids defects on the TQDs surface. In TQDs synthesis, octadecane is commonly used as a solvent, and ligands such as trioctylphosphine oxide, oleylamine, octadecylamine, ethylenediamine oleic acid, and 1-dodecanethiol are employed (Jain et al., 2020).

Chuang et al. (Chuang et al., 2014) reported the synthesis of CuInS_2_ with the structure of chalcopyrite at different [Cu]/[In] molar ratios by a solvothermal route, heating a solution (cuprous iodide and indium acetate dissolved in 1-dodecanethiol) using a Teflon-lined autoclave at 180 °C for 5 h and 30 min CuInS_2_ was coated with a ZnS shell by adding a shell stock solution (zinc stearate dissolved in a mixture of 1-dodecanethiol and octadecene) and heating at 200°C for 14 h. Synthesized CuInS_2_/ZnS presented high luminescence and a tunable emission wavelength from 550 to 616 nm by controlling the molar ratio of [Cu]/[In]. CuInS_2_/ZnS with a molar ratio of 1/2 of [Cu]/[In] presented a photoluminescence quantum yield (QY) of 81%. Orange- and red-emitting CuInS_2_/ZnS TQDs were used in the fabrication of WLEDs that exhibited high color rendering index values ∼90 and luminous efficacies of 36.7 Im W^−1^.

Li et al. (Li et al., 2020) prepared CuInS_2_/ZnS TQDs by the solvothermal method. The CuInS_2_ TQDs were synthesized using a Teflon-lined autoclave where a solution (cuprous iodide and indium acetate dissolved in 1-dodecanethiol) was heated at 180 °C for 6 h. After that, CuInS_2_ the TQDs were combined with a shell stock solution (zinc acetate dissolved in a mixture of 1-dodecanethiol, octadecene, and oleic acid) and heated at 200 °C for 14 h. ZnS shell formation on the CuInS_2_ TQDs allowed a QY increase up to 85%. The CuInS_2_ TQDs with [Cu]/[In] molar ratio of 1/2 were used to study the effect of the nucleation temperature (180°C), obtaining a core with good dispersion and an average size distribution of 2.89 nm. By controlling the [Cu]/[In] molar ratios and nucleation temperature, the CuInS_2_ TQDs exhibited a tunable emission wavelength from 651 to 775 nm. Synthesized CuInS_2_/ZnS TQDs with (Ba, Sr)_2_SiO_4_:Eu^2+^ phosphor as color converters, in combination with a blue GaN chip, were used to produce WLEDs that showed high color rendering index values (∼90) and a correlated color temperature of 4360 K.

### Hot-injection method

The hot-injection method is based on the addition of the cationic precursors in one step into an organic solvent at a high temperature (≥190°C). Usually, the process takes place in a reactor under a flow of nitrogen or argon to avoid the interference of oxygen species. The quick addition of precursors and the use of high temperatures promotes a burst nucleation and, afterwards, the growing of the particle, leading to the formation of homogeneous nanocrystals. Therefore, the broad or narrow distribution of nanoparticle size is dependent of a slow or fast injection of the precursors (de Mello Donegá et al., 2005; Ghorpade et al., 2014; Kulpa-Greszta et al., 2021).

Hu et al. (Hu et al., 2019) synthesized the CuGaS_2_/ZnS TQDs with relatively uniform sizes that exhibited a tunable emission wavelength from 520 to 619 nm by controlling the [Cu]/[Ga] molar ratios *via* the hot-injection method, where 1-dodecanethiol was used as a solvent, sulfur precursor, and ligand. The CuGaS_2_/ZnS TQDs were prepared with a shell stock solution (anhydrous zinc acetate and 1-octadecene were dissolved in a mixture of oleic acid and 1-dodecanethiol), which was injected into the reaction crude (cuprous iodide and gallium acetylacetonate dissolved in 1-dodecanethiol) at 250°C for 60 min. The CuGaS_2_/ZnS TQDs presented a full width at a half maximum of near 75 nm. A WLED was fabricated using yellow CuGaS_2_ TQDs by depositing the nanoparticles on a blue InGaN chip, exhibiting a luminous efficacy of 11.9 Im W^−1^.

Deng et al. (Deng et al., 2020) synthesized efficient green light emission CuInS_2_/ZnS TQDs by a hot-injection method at low temperature (130 °C) combined with a covering strategy of multilayer ZnS. Instead of the conventional dodecanethiol, they used S powder dissolved in oleylamine as the sulfur source for the covering process. The obtained CuInS_2_/ZnS showed a maximum QY ∼ 85%, a wavelength close to 530 nm, and they were employed in the fabrication of LEDs that exhibited an excellent external quantum efficiency (EQE) of 1.44%.

Wei et al. (Wei et al., 2020) obtained AgInS_2_/ZnS TQDs that exhibited a tunable emission wavelength and high QYs values (72%). The AgInS_2_ TQDs were synthesized by a hot-injection strategy and coated with a ZnS layer. The AgInS_2_/ZnS TQDs showed an average diameter of 2.5 nm with a homogeneous size distribution, generally with an orthorhombic chalcopyrite-type structure or tetragonal phases, which were related to the synthesis temperature. The AgInS_2_/ZnS TQDs were used in the preparation of LEDs that showed a highest EQE of 1.25%.

Li et al. (Li et al., 2023) prepared InP/ZnS QDs and AgInS_2_/ZnS TQDs by a one-pot hot-injection method. The AgInS_2_/ZnS TQDs were synthesized at different stoichiometric ratios of [Ag]/[In]. These TQDs exhibited wavelengths from 515 to 804 nm, large stocks shift (>100 nm), and QYs >60%. In turn, the synthesized InP/ZnS QDs presented a narrow PL peak width (40 nm), QYs >70%, and a widely tunable green emission wavelength. Finally, the combination of green-emitting InP/ZnS QDs with the AgInS_2_/ZnS TQDs (560–690 nm) and a blue GaN chip allowed the fabrication of warm WLEDs of high color quality, with luminous efficacy up to 75.2 lm W^− 1^ and a correlated color temperature of 3114K.

### Heating-up or non-injection method

Although the hot-injection method is a common synthetic strategy, it has some disadvantages when up-scaling the volume of the system is required. The main problems are the broad size distribution of nanoparticles (10–30 nm), low reproducibility (differences in morphology, element ratio, etc.), and the reaction cooling time. The heating-up method (also known as the non-injection method) is an alternative to the hot-injection method. The methodology is based on the addition of the precursors to a solvent at room temperature and a gradual heating up to obtain the monomers, the formation of the nuclei, and the obtention of the nanocrystal (Van Embden et al., 2015).

Park et al. (Park et al., 2015) synthesized different structures of CuInS_2_ TQDs (core, core/shell, and core/shell/shell) for the development of yellow, green, and WLEDs. The CuInS_2_ core TQDs were synthesized by mixing Cu- and In-oleate precursors with dodecanethiol as a sulfur source in 1-octadecene and heating the solution to 100°C for degassing and to 230°C for 30 min for core formation. The synthesis of the CuInS_2_/ZnS core/shell TQDs used the CuInS_2_ core TQDs synthesized in the first step, and to obtain the core/shell structure, a mixture of Zn-oleate precursor and dodecanethiol in octadecene was added, followed by heating to 250 °C for 7 h. The CuInS_2_/ZnS/ZnS core/shell/shell TQDs were obtained by using the synthesized CuInS_2_/ZnS TQDs and repeating the second step conditions. An increment in the particle size was observed in every stage of the synthesis, with a particle size of the core of 2.5 nm, a core/shell of 3.5 nm, and a core/shell/shell of 4.3 nm. A hypsochromic shift from the CuInS_2_ TQDs (670 nm, red emission) to CuInS_2_/ZnS TQDs (576 nm, yellow emission) and CuInS_2_/ZnS/ZnS TQDs (559 nm, yellow–green emission) was observed. Quantum efficiency of the TQDs increased from 31.7% for the core to 80.0% for the core/shell/shell structures. The CuInS_2_/ZnS/ZnS TQDs structures were used in a WLED, obtaining a luminous efficacy of 80.3 lm W^−1^.

### Thermal decomposition method

Generally, nanoparticle synthesis by thermal decomposition consists of the thermal degradation of organosulfur precursors in organic solvents in the presence of surfactants for stability of the nanoparticle. The common precursor used for AgInS_2_ is AgIn(S_2_CN(C_2_H_5_)_2_)_4_. However, the toxicity of diethyldithiocarbamate has limited its use in nanoparticle synthesis for LED applications (Maji, 2022).

**FIGURE 1:**
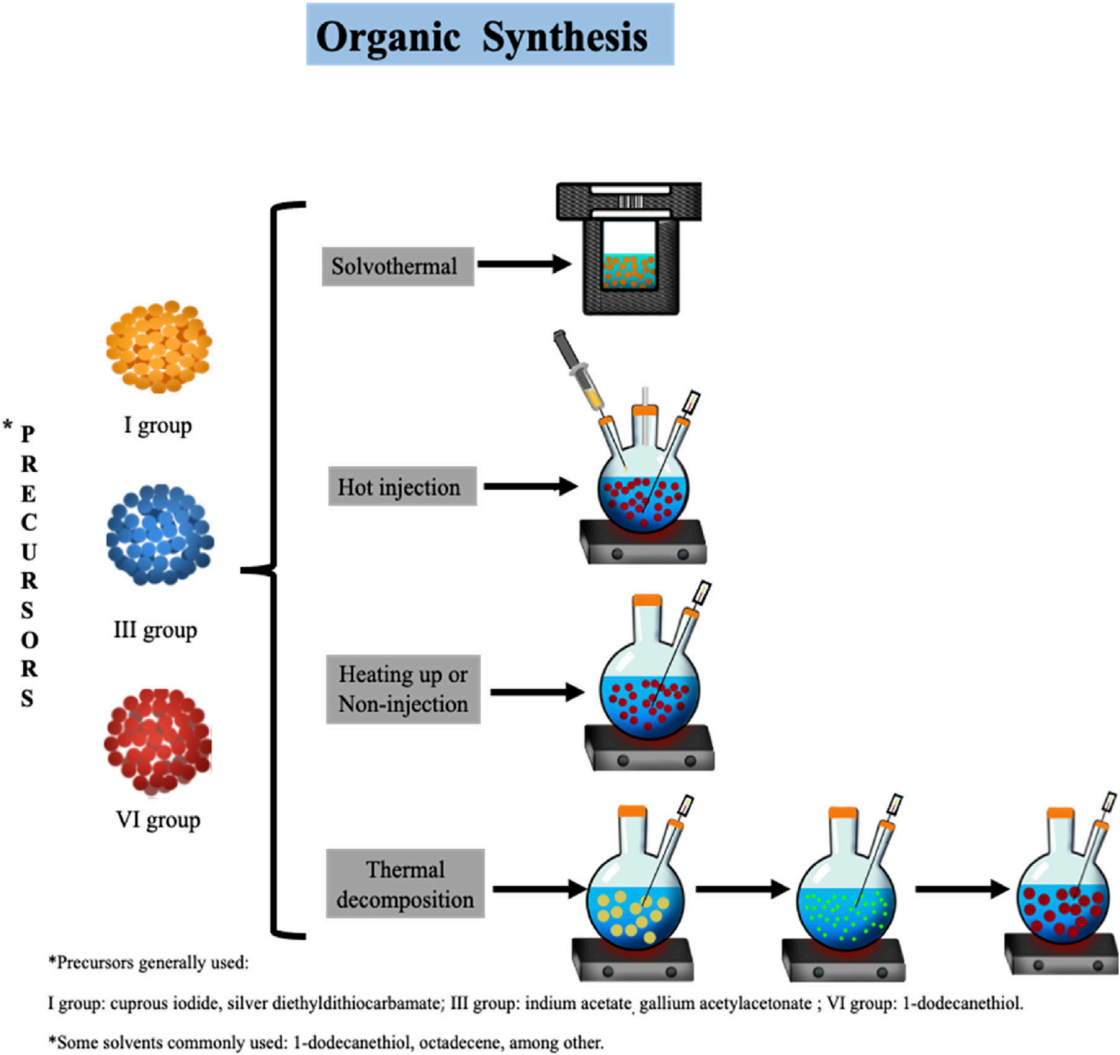
Organic synthesis of ternary quantum dots by diverse methods: solvothermal, hot injection, heating up, and thermal decomposition.

Chung et al. (Chung et al., 2014) synthesized Zn-doped AgInS_2_ TQDs with spherical shape and size <7 nm by thermal decomposition. The authors replaced the diethyldithiocarbamate-based precursor for silver nitrate, indium acetate, and 1-dodecanethiol. The precursors were dissolved in a mixture of 1-octadecene, trioctylphosphine, and oleic acid, which were used as surfactants and heated to 100°C, followed by the addition of sulfur powder and zinc stearate for the obtention of the shell at 130°C. The authors described the phases corresponding to an orthorhombic structure for the AgInS_2_ TQDs, and after the incorporation of Zn ions, a cubic structure was observed, thus suggesting that a phase transition took place. The Zn-doped AgInS_2_ TQDs exhibited an improvement in the QYs in the range from 3% to 15% and emission wavelengths in the range from 644 to 539 nm. A WLED was fabricated by combining a 380 nm UV LED, Zn-doped AgInS_2_ TQDs (618 nm) and carbon dots, obtaining a color rendering index of 96.2 (see Figure 1; Table 1).

**TABLE 1:** Overview of organic and aqueous synthesis of TQDs, their properties, and LEDs parameters.

Particle (core/shell)	Synthesis method	Physicochemical properties	Device fabrication and optoelectronic characteristics	References
CuInS_2_/ZnS	Organic synthesis**/**Solvothermal	*Tunable emission wavelength from 550 to 616 nm by adjusting the [Cu]/[In] molar ratios	Orange- and red-emitting CuInS_2_/ZnS TQDs were used in the fabrication of WLEDs	Chuang et al. (2014)
		*Molar ratio of 1/2 of [Cu]/[In] presented QY 81%.	⁃ Luminous efficacies: 36.7 Im W^−1^	
			⁃ Color rendering index: ∼90	
CuInS_2_/ZnS	Organic synthesis**/**Solvothermal	*Emission bands were tuned from 651 to 775 nm by controlling the [Cu]/[In] molar ratios	* CuInS_2_/ZnS TQDs with (Ba, Sr)_2_SiO_4_:Eu^2+^ phosphor and a blue GaN chip were used for fabricated WLEDs	Li et al. (2020)
		*After shell formation on CuInS_2_ TQDs allowed QY increase to 85%	⁃ Color rendering index value: ∼90	
			⁃ Correlated color temperature: 4360 K	
CuGaS_2_/ZnS	Organic synthesis**/**Hot-injection	*Emission bands were tuned from 520 to 619 nm by controlling the [Cu]/[Ga] molar ratio	A WLED was fabricated using yellow CuGaS_2_ TQDs dropped on blue InGaN chip	Hu et al. (2019)
		*Narrow full-width at half maximum wavelength of ∼75 nm	⁃ Luminous efficacy: 11.9 Im W^−1^	
CuInS_2_/ZnS	Organic synthesis/Hot-injection	*A maximum QY ∼ 85% and wavelength close to 530 nm	CuInS_2_/ZnS TQDs were used in the fabrication of LEDs	Deng et al. (2020)
			⁃ EQE of 1.44%	
AgInS_2_/ZnS	Organic synthesis/Hot-injection	* QYs ∼72%	AgInS_2_/ZnS TQDs were used in the preparation of LEDs	Wei et al. (2020)
		*Average diameter ∼2.5 nm	⁃ EQE of 1.25%	
AgInS_2_/ZnS	Organic synthesis/Hot-injection	*Exhibited wavelengths from 515 to 804 nm	Combination of green-emitting InP/ZnS with AgInS_2_/ZnS TQDs (560–690 nm) and a blue GaN chip were used to fabricate warm WLEDs	Li et al. (2023)
		*Large Stokes shift (>100 nm)	Luminous efficacy: 75.2 Im W^−1^	
		*QYs >60%	Correlated color temperature: 3114 K	
CuInS_2_/ZnS/ZnS	Organic synthesis/Heating up or non-injection	* Particle size of 4.3 nm	CuInS_2_/ZnS/ZnS TQDs were used for a WLED	Park et al. (2015)
		*A peak wavelength of 559 nm, yellow–green emission	⁃ Luminous efficiencies: 80.3 Im W^−1^	
		*Quantum efficiency of the TQDs increased from 31.7% for core to 80.0% for core/shell/shell structures		
Zn-doped AgInS_2_	Organic synthesis**/**Thermal decomposition	* Size <7 nm	WLED was fabricated by combining a 380 nm UV LED, Zn-doped AgInS_2_ TQDs (618 nm), and carbon dots	Chung et al. (2014)
		*An improvement in the QYs from 3% to 15%	⁃ Color rendering index: 96.2	
		*Emission wavelength from 644 to 539 nm		
AgInS_2_/ZnS	Aqueous synthesis/Hydrothermal	*An improvement in QYs from 21.6% to 45%	The obtained AgInS_2_/ZnS TQDs were combined with Lu_3_Al_5_O1_2_:Ce^3+^ and used in the fabrication of a WLED	Chen T. et al. (2019)
			⁃ Luminous efficiency: 77.98 Im W^−1^	
			⁃ Color rendering index: 85	
			⁃ Correlated color temperature: 6215 K	
MPA-capped CuInS_2_/ZnS	Aqueous synthesis/Hydrothermal	*Spherical shape	—	Jain et al. (2022)
		*QYs values from 6.95% to 17.19%		
CuInS_2_/ZnS	Aqueous synthesis**/**Microwave assisted	*Average diameter of 4.8 nm	CuInS_2_/ZnS/PVP were used as nanocomposite emitters of green and red and converters of colors by combination of a blue LED chip	Ji et al. (2016)
		*The molar ratio of Cu/In in optimal conditions reached a maximum QYs of 43%		
		*Tunable emission wavelength from 543 to 700 nm by controlling the [Cu]/[In] molar ratios		
AgInS_2_/ZnS	Aqueous synthesis**/**Microwave assisted	*Average size 3.07 nm	AgInS_2_/ZnS TQDs were used in the preparation of warm WLEDs	Su et al. (2020)
		*A shift in the emission wavelength from 540 to 622 nm by adjusting molar ratio of [Ag]/[In]	⁃ Color rendering index: 87.5	
		*Reached a maximum QY of 58.27%	⁃ Correlative color temperature: 3669 K	

### Aqueous synthesis

The synthesis of TQDs in water has the advantage of being more environmentally friendly compared to synthesis in organic solvents. Along with the solvent used, reagents (precursors and ligands) present lower toxicity, and the synthesis protocols are easier to apply. Normally, aqueous synthesis uses inorganic metal precursors and thiourea or sodium sulfide as the sulfur source due to their high solubility in water. Additionally, some strategies do not require the use of an inert atmosphere (Jain et al., 2020). The main strategies for TQDs aqueous synthesis include hydrothermal and microwave-assisted methods.

### Hydrothermal method

Typically, hydrothermal methods used for the synthesis of nanoparticles consist of heating an aqueous mixture of precursors in a sealed container to a temperature higher than the water boiling point (Hu and Zhu, 2015; Huang et al., 2019).

Chen et al. (Chen W. et al., 2019) synthesized highly fluorescent AgInS_2_/ZnS TQDs by a hydrothermal method using AgNO_3_ and In(NO_3_)_3_ as cation precursors, Na_2_S as the sulfur source, and sodium citrate and glutathione as ligands. The precursor mixture was heated in an autoclave at 110°C for 7 h to obtain the core. The shell was obtained by the addition of Zn(OAc)_2_ as the Zn^2+^ precursor and placing the reaction in an autoclave at 90°C for 5 h. The resulting core/shell TQDs showed an improvement in the QYs value from 21.6% to 45%. The obtained AgInS_2_/ZnS TQDs were combined with Lu_3_Al_5_O_12_:Ce^3+^ and used in the fabrication of a WLED, which exhibited good properties: a luminous efficiency of 77.98 Im W^−1^, correlated color temperature of 6215 K, and color rendering index of 85.

Jain et al. (Jain et al., 2022) synthesized aqueous mercaptopropionic acid (MPA)-capped CuInS_2_/ZnS TQDs through the hydrothermal method. To obtain the core structure, an aqueous solution of CuCl_2_, InCl_3_, thiourea, and MPA was prepared at pH 11.3. This solution was heated in an autoclave at 150 °C for 21 h and then cooled to room temperature. The ZnS shell was grown on the core structure by the addition of Zn(OAc)_2_ as the Zn^2+^ precursor and Na_2_S as the sulfur source. The obtained CuInS_2_/ZnS TQDs presented a spherical shape and reached QYs values from 6.95% to 17.19%.

### Microwave-assisted aqueous method

This technique is based on focused irradiation to generate intense heating over polar substances, and several microwave strategies allow to reduce the reaction time for TQDs synthesis (≤20 min) compared to the organic and hydrothermal methods (Janis et al*.,* 2019). Generally, microwave-assisted methods are highly reproducible in the production of QDs with a narrow size distribution. In a typical synthesis assisted by microwaves, the reaction temperature is close to the water boiling point (90°C–110 °C). As in the hydrothermal methods, the metallic inorganic precursors are mixed with a stabilizing agent in water, in a controlled pH (usually basic), and subsequently a chalcogenide salt is added (Muñoz et al., 2021).

Ji et al. (Ji et al., 2016) synthesized CuInS_2_/ZnS TQDs embedded in solid polyvinylpyrrolidone (PVP) in an aqueous phase at 95°C for 20 min through assisted microwave synthesis in order to improve their thermostability and the photostability and intensity of the photoluminescence. The authors reported CuInS_2_ and CuInS_2_/ZnS TQDs with spherical shape, uniform morphology, and average diameters of 3.2 and 4.8 nm, respectively. The obtained CuInS_2_/ZnS TQDs exhibited a maximum QYs of 43% and a tunable emission wavelength at a wide range from 543 to 700 nm by controlling the [Cu]/[In] molar ratios. The CuInS_2_/ZnS TQDs embedded in solid PVP were used as nanocomposite emitters of green and red and converters of colors by the combination with a blue LED chip.

**FIGURE 2:**
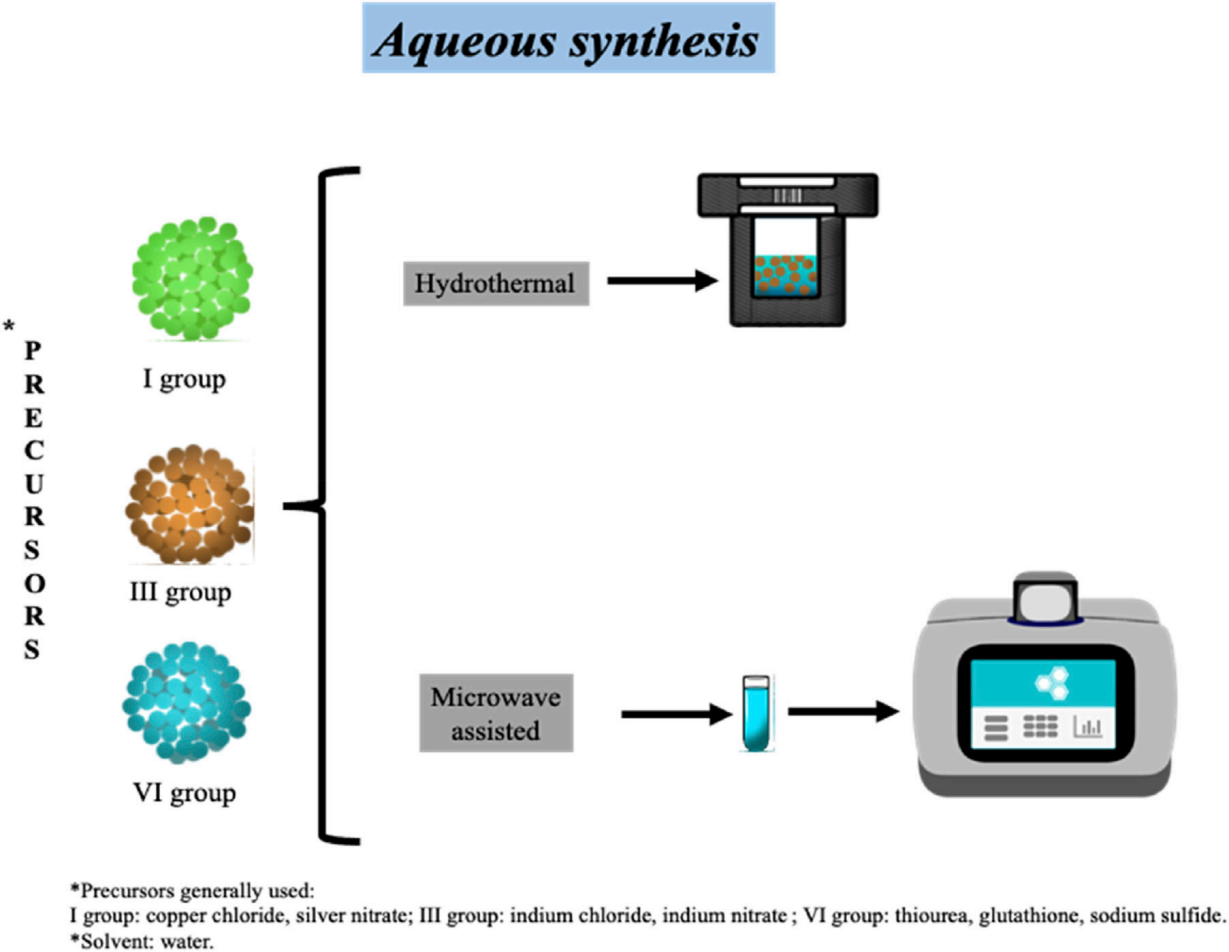
Aqueous synthesis of ternary quantum dots by hydrothermal and microwave-assisted methods.

Su et al. (Su et al., 2020) described the preparation of AgInS_2_/ZnS TQDs in an aqueous phase by microwave-assisted synthesis in two steps, one for core obtention (95 °C, 1 h) and the other for shell synthesis (95 °C, 10 min). Subsequently, the TQDs were embedded on polyacrylamide hydrogels and combined with blue InGaN chips to fabricate warm WLEDs. The obtained TQDs showed an average size of 3.07 nm and a shift in the emission wavelength from 540 to 622 nm by adjusting the molar ratio of [Ag]/[In] from 0.05 to 0.5, reaching a maximum QYs of 58.27% when a molar ratio of 0.1 was used. The synthesized AgInS_2_/ZnS TQDs were used for WLEDs fabrication, showing a high color rendering index of 87.5 and a correlative color temperature of 3669 K, hence indicating that they are competitive materials for color conversion in warm WLEDs (see Figure 2; Table 1).

## Physicochemical properties of TQDs and LEDs parameters

The study of the physical and chemical properties of TQDs is relevant in LEDs fabrication, because they influence the characteristics and performance of the final product. In LEDs, TQDs have several useful properties such as i) tunable capacity of absorption and emission length by controlling the size, shape, structure, and precursors stoichiometric ratios; ii) QY that is coupled to the EQE of the LEDs; iii) good photostability (Zhong et al., 2011; Bai et al., 2016); iv) large stokes shifts; v) long photoluminescence lifetime; and vi) low toxicity. Therefore, efforts have been made to generate new LEDs with proper characteristics (adequate emitting color, EQE, long lifetime, color purity, etc.) based on TQDs (Mei et al., 2018; Bang et al., 2021).

### TQDs physicochemical properties

An advantage of TQDs in LEDs fabrication is that they can be tuned in any color of the spectra, and therefore different LEDs can be designed using the same material. The TQDs emission wavelength is related to the band gap of the nanomaterial, and this property (commonly found in conductor and semiconductor materials such as TQDs and QDs) is defined as the energy range between the valence band and the conduction band; it is different in bulk and nanoparticle materials. For instance, AgInS_2_ and CuInS_2_ exhibit a band gap of 1.87 and 1.27 eV, respectively, but when the materials present nanometric scales, the band gap value differs (from 1.6 to 1.9 for CuInS_2_ and from 2.3 to 3.1 for AgInS_2_) (Raevskaya et al., 2017; Xia et al., 2018).

Usually, at the nanometric scale of TQDs, the band gap energy increases when the electron–hole pair is more confined. Therefore, a decrease in the TQDs size will result in nanoparticles that emit near the ultraviolet spectra, and an increase in the TQDs size will produce nanoparticles that emit near the infrared spectra (Singh et al., 2021).

Xia et al. (Xia et al., 2018) synthesized CuInS_2_ with different sizes from 2.1 to 6.1 nm, obtaining different emission colors from the same material (violet and red, respectively). The band gap changed from 1.9 to 1.6 eV when the nanoparticles size increased. Mir et al. (Mir et al., 2018) observed the same phenomenon in AgInS_2_ TQDs, obtaining nanoparticles with tunable band gap from 3.1 to 2.3 eV with different sizes and emission spectra. Thus, the color emitted by the LED will correspond to the band gap energy of the semiconductor integrated in it (Singh et al., 2021).

A second optical property of TQDs is QY. This is an essential spectroscopic parameter of fluorescent materials, which measures the ratio between photons emitted and photons absorbed. QY is related to the structure and stability of the nanocrystal and can be controlled in the synthesis protocol, specifically on the surface structure (Sadeghi et al., 2018; Fries and Reineke, 2019). Different strategies have been described to enhance the TQDs QY, but for LED applications the most useful is the addition of a shell to the TQDs core. In TQDs synthesis, it is common that not all cations react with the chalcogenide, and in nanoparticle obtention, the species relocate on the surface. These species, also called traps, do not allow a proper recombination process, reducing the QY (Zhang et al., 2021). By passivating the TQDs surface, the QY improves for TQDs, and the shell used is ZnS. The ZnS shell is able to enhance the QY of CuInS_2_ from 3.2% to 81%, according to Chuang et al. (Chuang et al., 2014). Wei et al. (Wei et al., 2020) described an increase in the QY of AgInS_2_ from 57% to 72% by adding a ZnS shell.

Table 1 depicts QY values of different TQDs, and it is well-known that QY values are higher when an organic solvent is used. This happens because the organic synthesis occurs at higher temperature, promoting the formation of more homogeneous nanocrystals with fewer surface defects. On the other hand, in aqueous synthesis more traps are located on the surface, allowing non-emitting transitions and decreasing the fluorescence of the materials (Pu et al., 2018).

Concentration of precursors is another factor to be considered in the synthesis of TQDs. According to different authors, the Ag/In and Cu/In ratios have a bathochromic effect when the group I cations are in a 1/1 stoichiometry. For instance, Huong et al. synthesized AgInS_2_ TQDs using a molar ratio of Ag/In from 1/10 to 1/1, showing a change in the emission from 590 nm at 1/10 ratio to 640 nm at 1/2 ratio and 745 nm at 1/1 ratio (Huong et al., 2022).

The same spectroscopic behavior was described by Wei et al. in AgInS_2_ TQDs synthesized by the hot-injection method. The Ag/In ratios were evaluated from 1/5 to 1/1, showing a bathochromic effect when the Ag^+^ and the In^3+^ concentrations were equal. The TQDs were shown to exhibit a maximum in fluorescence at 603 nm in a 1/5 ratio, 700 nm in a 1/3 ratio, and 868 nm in a 1/1 ratio (Wei et al., 2020). Band gap and emission maximum wavelength values were attributed to the interaction between the 3p orbital from sulfur and 4d orbital from silver; therefore, in a lower Ag^+^ concentration the interaction is lowered, and more energy is required to excite the electron to the conduction band obtaining the emission at lower wavelengths (Song et al., 2016). The CuInS_2_ TQDs show the same Cu/In ratio pattern. Li et al. synthesized CuInS_2_ TQDs from 1/3 to 1/0.5 Cu/In ratios, and the emission maximum changed from 638 to 714 nm (Li et al., 2020).

Other physicochemical properties can directly affect the performance of LED and WLED based on TQDs properties, and for a proper selection of semiconductors the Stokes shift and the lifetime of the fluorescence are parameters to be considered. The Stokes shift is defined as the difference in energy (in meV) between the maximum absorption wavelength and the maximum wavelength of the fluorescence emission. TQDs present a large Stokes shift, which improves the efficiency of QLED and WLED (Chan et al., 2021). CuInS_2_ exhibits a Stokes shift in the range from 200 to 500 meV and AgInS_2_ in the range from 300 to 1,000 meV, which are greater than the ones presented by some organic molecules used on OLED (Baimuratov et al., 2019).

Photoluminescence lifetime in semiconductors refers to the time that the electron remains in the conduction band before it returns to the valence band. Several organic compounds exhibit lifetimes of 10 nanoseconds, and carbon dots have lifetimes ≤20 ns, while TQDs display values of at least 100 ns (Hutton et al., 2017; Liu et al., 2020). The CuInS_2_ photoluminescence lifetime is in the range from 100 to 300 ns, and AgInS_2_ shows a photoluminescence lifetime in the range from 110 to 450 ns. High fluorescence lifetimes decrease the photobleaching of materials, thus allowing to achieve greater device lifetimes. These high values in the Stokes shift and photoluminescence lifetime make TQD an excellent option to use in the design of LED and WLED (Soares et al., 2020; Adeleye et al., 2021).

The success of QDs in LEDs devices is due to their high QY, stability, and relatively easy synthetic procedures. However, they generally include Cd-Pb precursors in the QDs (such as CdSe, CdS, CdTe, PbSe, and PbS) that have a negative impact on the environment (Kurshanov et al., 2018). The Environmental Protection Agency classifies cadmium in the group B I (probably carcinogenic to humans) and has established maximum contaminant levels in drinking water (5 μg L^-1^) (Agency for Toxic Substances and Disease Registry, 2010). Lead is classified in group B II (probably carcinogenic to humans) (U.S. Environmental Protection Agency, 2013), and the maximum level for bottled water is 5 μg L^-1^ (U.S. Food and Drug Administration, 2009). In this regard, TQDs are free of toxic elements, and this attractive feature, together with their broadly tunable optical properties, make them very suitable materials for implementation in LEDs (Chen et al., 2018).

### LEDs parameters

According to their use, LEDs performance can be measured in electrical (external quantum efficiency) and optical (luminous efficacy, color rendering index, etc.) parameters, which are dependent on TQDs properties and LEDs configuration.

LEDs are devices that are able to emit light by converting the electric energy in an electroluminescence process. EQE is a ratio of the number of emitted photons and the electrons applied through the LED, and in this sense, a higher %EQE value means a more efficient LED (Roedel, 2001; Plis et al., 2011).

One of the main optical LEDs parameters is the luminous efficiency, which is defined by the relation between the luminous flux and the power. This relation determines how well a light source produces visible light (Xu and Chen, 2019). In WLEDs, in addition to the luminous efficiency, it is necessary to know the quality of white light, which is measured by the color rendering index (CRI) and color appearance, defined by the proper correlated color temperature (CCT), and expressed in degrees Kelvin (Xu, 2019; Kalani and Kalani, 2021).

## Design of light-emitting diodes using ternary quantum dots

The capacity of tuning the emission wavelength of QDs by adjusting the nanoparticle size allows to obtain LEDs in the range from UV to IR, and for binary materials based on Cd (II) (such as CdTe, CdS, and CdSe), the optical properties have been widely explored. Nevertheless, there is a need to substitute these materials due to their high toxicity, and their ternary analogues such as AgInS_2_, CuInS_2_, and CuInSe_2_ represent a valid alternative (Oluwafemi et al., 2021).

**FIGURE 3:**
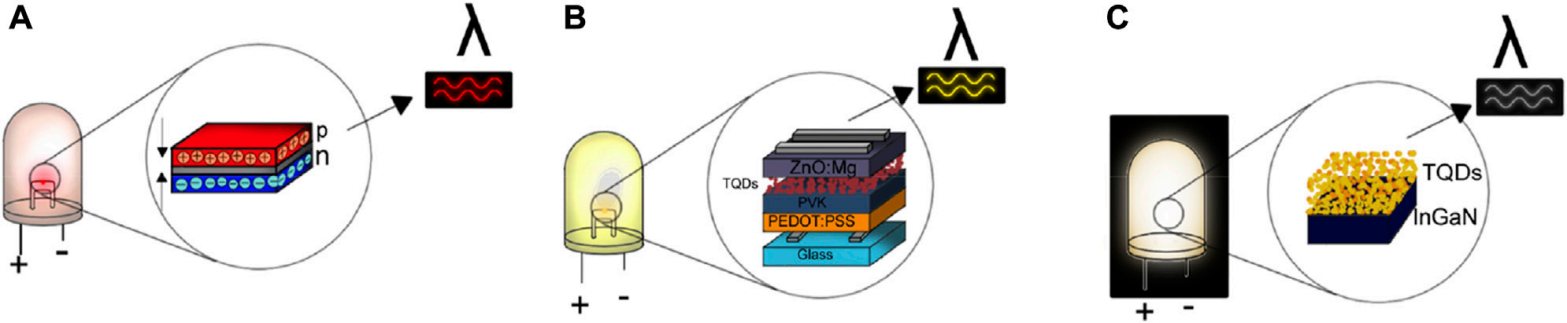
Design of LEDs based on TQDs: **(A)** classical configuration, **(B)** example of modern configuration, **(C)** WLEDs.

The general structure of an LED requires the conjunction of three semiconductor-based layers that are called the n-type, p-type, and the mid-layer, also called the active region. The n-type layer has an excess of electrons, and it is connected to the negative terminal of the LED, whereas the p-type layer has a deficiency in electrons and is hole-containing, being connected to the positive terminal of the LED (Figure 3A). When a current is applied, the electrons in the n-type layer and the holes from the p-type layer migrate towards the active region, where a recombination process occurs, resulting in light emission (electroluminescence). In semiconductors, the light emitted depends on the band gap of the material, and for TQDs, the band gap is determined by the particle size (Yuan et al., 2021). To fabricate LEDs, the layers can be arranged in either of two principals set-ups. In the first set-up, it is possible to use the same semiconductor material and incorporate impurities (ions) in one layer to obtain the electron-rich and hole-rich layers (p-n homojunction). A second approach is called the p-n heterojunction, where the fabrication of the QLED requires the use of two different semiconductors, one for the p-type layer and one for the n-type layer. Modern configurations include different layers such as the hole-injection layer, hole-transport layer, electron-transport layer, electron-injection layer, and emission layer (Bose et al., 2014). For LEDs using TQDs, the layers correspond to a heterojunction.

The performance of QLED is affected by the film thickness and uniformity of the mid-layer. There are different methodologies to obtain an adequate film between the carrier layers of the QLED, such as transfer printing, inkjet printing, mist deposition, and the Langmuir–Blodgett technique, but the main strategy used for TQDs is spin coating (Zhang et al., 2018).

The spin-coating method consists of diluting TQDs in a solvent, and this solution is dispersed on the surface of the layer where TQDs will be deposited. Subsequently, the wafer is rotated using centrifugal force until the solvent is evaporated and the film is deposited (Zhao et al., 2021). The layer thickness of the obtained TQDs is in the nanometric or micrometric scale, although a lower thickness increases the efficiency and brightness of the LED. The layer thickness is determined by the viscosity of the solution, rotation speed, surface tension, and concentration of the TQDs (Shmshad et al., 2019).

Most of the articles cited in this review describe spin coating as the strategy used for deposition of TQDs. However, this step has not been fully exploited, and only a few systems are completely described. Motomura et al. (Motomura et al., 2020) used the spin coating method with a solution of AgInS_2_/GaS_2_ TQDs (3 mg mL^-1^, dissolved in chloroform) over the electron injection layer of ZnO nanoparticles, resulting in the formation of multilayer TQDs with 15 nm thickness and %EQE of 0.54% when combined with tris(2,4,6-trimethyl-3-(pyridin-3-yl)phenyl)borane.

Binary semiconductor nanoparticles such as CdSe/CdS have also been evaluated in spin-coated strategies. Sayevich et al. (Sayevich et al., 2021) employed a procedure where 10 mg mL^-1^ of QDs were dispersed in octane as a solvent, after which the nanoparticles were spin-coated over PEDOT:PSS at 2000 rpm for 30 s and the system was heated to 80°C for 10 min, resulting in the formation of a mid-layer with 25 nm thickness. The %EQE of the resulting device was 0.43%.

Although film deposition is a crucial step in QLED design and performance, this has not been fully studied, in terms of rotation speed, concentration of TQDs, thickness, and obtained %EQE. Strategies such as electrohydrodynamic jet spraying and inkjet printing have been shown to be adequate strategies for layer formation in binary QDs for QLED. Nevertheless, the behavior of TQDs might be different; therefore, further research is required in this area (Xiong et al., 2019).

Deng et al. (Deng et al., 2020), fabricated a mid-layer using spin coating to deposit CuInS_2_/ZnS and disperse it in n-octane at a concentration of 20 mg mL^-1^, at 3,000 rpm, and 70°C for 40 s.

Different configurations of LEDs are described in the literature, and TQDs are used as a mid-layer or mid-layer additive (Figure 3B). Lv et al. (Lv and Liang, 2017) designed an LED by using (poly(3,4-ethylenedioxythiophene) poly styrenesulfonate (PEDOT:PSS) as the hole-transport layer, 2,2′,2"-(1,3,5-Benzinetriyl)-tris(1-phenyl-1-H-benzimidazole) as the electron-transport layer connected to a LiF/Al cathode, and a layer of AgInS_2_ TQDs for the active region. The AgInS_2_ TQDs used in the LED presented a diameter of 4.8 nm and a maximum of fluorescence at 730 nm, thus producing a red light-emitting diode.

Wei et al. (Wei et al., 2020) developed an LED using AgInS_2_/ZnS core/shell TQDs. For the construction of the LED, indium–tin oxide was used as a glass substrate, where a layer of PEDOT:PSS was deposited as the hole-transfer layer, followed by a layer of Poly[bis(4-phenyl) (4-butylphenyl)amine] as a second hole-transfer layer. The active region was obtained by depositing AgInS_2_/ZnS over the hole-transfer layer surface. The electron-transfer layer was composed of ZnO:Mg particles, and an aluminum film was used as a cathode. AgInS_2_/ZnS QDs exhibited an average diameter size of 4.9 nm and a maximum emission at 666 nm, with a full width at half maximum of 122 nm. The obtained LED exhibited an external quantum efficiency of 1.25%.

Deng et al. (Deng et al., 2020) developed a green light-emitting diode using indium–tin oxide as a substrate, PEDOT:PSS and Poly(9-vinylcarbazole) as the hole-transfer layer, CuInS_2_/ZnS core/shell QDs as the active region, ZnO nanoparticles as the electron-transfer layer, and Ag film as a cathode. The structure allowed to reduce the Auger recombination, which is a process where the excess energy is not converted to photons, but rather the electrons are transferred to higher energy states (Jung et al., 2021). The obtained EQE was 1.44%, which is a promising value according to the author.

Core/shell/shell structures can also be used in LED fabrication. Ye et al. (Ye et al., 2020) designed an LED based on CuInS_2_/ZnS/ZnS QDs, with the structure ITO/PEDOT:PSS/Poly(9,9-dioctylfluorene-alt-N-(4-sec-butylphenyl)-diphenylamine)/TQDs/PEI (polyethyleneimine)/ZnO/Ag. The core/shell/shell structure exhibited a high QY (76%), allowing to obtain an LED with good performance. Moreover, by adding a layer of PEI, there was an enhancement in the efficiency of the LED compared to a core/shell structure (EQE 1.56%).

Not only can TQDs be used for monochromatic LEDs, but they can also be employed as components in the fabrication of polychromatic LEDs or WLEDs (Figure 3C). Generally, a WLED is formed by a mixture of different color materials in the mid-layer, particularly blue, green, and red, where the color blue is an LED chip from InGaN, the color green is an LED chip from a phosphor compound, and the color red is an LED chip from TQDs (such as the materials with primary colors red, yellow, and blue). TQDs are used as additives in the mid-layer in a prefabricated LED chip (InGaN) (Liu et al., 2022).

Su et al. (Su et al., 2020) synthesized yellow AgInS_2_/ZnS QDs and applied a film over blue InGaN chips to obtain WLEDs. The authors also used red and green AgInS_2_/ZNs QDs films over blue InGaN chips to obtain WLEDs. The produced WLEDs exhibited a color rendering index of 75.5 and 87.5, respectively, which were considered promising materials. In another study, Chen et al. (Chen W. et al., 2019) designed a WLED using a blue InGaN chip, yellow AgInS_2_/ZnS QDs, and Lu_3_Al_5_O_12_:Ce3^+^ phosphor as source of green light. The resulting LED exhibited an increment of the CRI of 85.0% by adding the green light, compared to the QDs/InGaN mid-layer.

Hu et al. (Hu et al., 2022) obtained AgInS_2_/ZnS TQDs with a QY of 33.1% by a novel microwave organic synthesis. The resulting TQDs emitted orange–yellow light under UV light and were used in the fabrication of WLEDs. AgInS_2_/ZnS were mixed with a Y_3_Al_5-x_Ga_x_O_12_:Ce^3+^ phosphor compound and silicon, and the mixture was dispensed over a blue LED chip. The system was cured at 150°C for 1 h to obtain the WLED, which showed a luminous efficacy of 58.83 lm/W and a color rendering index of 87.6.

Different phosphor compounds can be used for WLED development. Chen et al. (Chen et al., 2023) used the europium-based phosphor compound (Ba, Sr)Si_2_O_2_N_2_:Eu^2+^ in a mixture with orange AgInS_2_/ZnS TQDs, which was deposited over a blue light LED. Silicon was added, and the mixture was cured under UV light. The TQDs exhibited a %QY of 35.66% and the resulting WLED presented a color rendering index of 87.7 and a luminous efficiency of 80.13 lm/W, which was attributed to the shell of the TQD.

Using the same structure as in the previous article, Dong et al. (Dong et al., 2019) incorporated red-emitting CuInS_2_/ZnS (65.07% QY) in a system in the presence of Y_3_Al_5_O_12_:Ce^3+^. These compounds were mixed with silicone to obtain a film, which was deposited and cured over an LED blue chip to obtain a WLED. The resulting device had a corelated color temperature of 3415 K.

## Conclusion and perspectives

TQDs are novel nanoparticles with unique physicochemical properties that are adequate in the design of LEDs. This review is focused on the synthesis, physicochemical properties, and use of TQDs in the fabrication of LEDs.

Different strategies have been developed for TQDs synthesis in organic and aqueous media, allowing to obtain nanoparticles with promising physicochemical properties. So far, the spectroscopic parameters of TQDs (such as QY and FWHM) have not reached the binary QDs values; therefore, further research is needed to improve them. Non-etheless, these nanomaterials have the advantage of using precursors with less toxicity compared to Cd- and Pb-based QDs. Optical properties and LEDs parameters are influenced by the size, composition, and solvent used in TQDs synthesis; thus, the critical point to be evaluated is the synthesis strategy and conditions. For instance, some parameters such as the QY (and therefore EQE) can be improved by changing the ratio of the group I and group III concentrations or by coating the core with a ZnS shell.

For TQD application into LEDs, the required parameters (such as color and EQE) in the device must be established before synthesizing the TQDs. The CuInS_2_ and AgInS_2_ TQDs are excellent candidates for efficient LEDs and WLEDs, and different designs have been proposed using TQDs in the mid-layer with adequate performance.

The performance of TQDs as light-emitting diodes can be improved in the future by focusing mainly on increasing the QY and reducing FWHM in TQDs synthesis, as well as increasing the EQE in the LEDs design and even exploring other materials, such as substituting Ag^+^ for Cu^+^ or Au^+^, In^3+^ for Ga^3+^ or Al^3+^, and S^2-^ for Se^2-^.
